# Cell-bricks based injectable niche guided persistent ectopic chondrogenesis of bone marrow-derived mesenchymal stem cells and enabled nasal augmentation

**DOI:** 10.1186/s13287-015-0006-4

**Published:** 2015-03-10

**Authors:** Ruikai Ba, Jianhua Wei, Man Li, Xiaobing Cheng, Yimin Zhao, Wei Wu

**Affiliations:** State Key Laboratory of Military Stomatology, Department of Prosthodontics, School of Stomatology, the Fourth Military Medical University, Changle West Road 145, Xi’an, China; State Key Laboratory of Military Stomatology, Department of Maxillofacial Surgery, School of Stomatology, the Fourth Military Medical University, Changle West Road 145, Xi’an, China

## Abstract

**Introduction:**

Developing cartilage constructs with injectability, appropriate matrix composition and persistent cartilaginous phenotype remains an enduring challenge in cartilage repair. Bone marrow derived mesenchymal stem cells (BMSCs) have chondrogenic potential. Current approaches to drive their chondrogenic differentiation require extensive cell manipulation ex vivo and using exogenous growth factors. However, preventing hypertrophic transition of BMSCs in vivo and maintaining persistent chondrogenesis remain bottlenecks in clinical application. This study aimed to develop completely biological, injectable constructs to generate cartilage by co-transplanting chondrocyte and BMSCs.

**Methods:**

We fabricated fragmented chondrocyte macroaggregate (cell bricks) and mixed them with platelet rich plasma (PRP); BMSCs were mixed into the above constructs, allowed to clot and then subcutaneously injected into nude mice. Gross morphology observation, histological and immunohistochemical assay, immunofluorescence assay, biochemical analysis and gene expression analysis were used to compare the properties of BMSC-cell bricks-PRP complex with BMSC in PRP or BMSC/chondrocytes in PRP.

**Results:**

The constructs of BMSCs-cell bricks-PRP that were subcutaneously injected resulted in persistent chondrogenesis with appropriate morphology, adequate central nutritional perfusion without central necrosis or ossification, and further augmented nasal dorsum without obvious contraction and deformation.

**Conclusions:**

We concluded that cell bricks-enriched PRP clotting provides an autologous substance derived niche for chondrogenic differentiation of BMSCs *in vivo*, which suggests that such an injectable, completely biological system is a suitable stem cell carrier for micro-invasive cartilage repair.

## Introduction

Chondrogenesis is the process by which cartilage is formed from condensed mesenchyme tissue. During the process, condensing mesenchyme expresses various extracellular matrix (ECM) and signaling molecules, which drives the compaction of mesenchymal cells, shaping of the condensations and chondrogenic differentiation of cells [1,2]. For therapeutic cartilage regeneration, harvesting bone marrow-derived mesenchymal stem cells (BMSCs) and reconstituting them using three-dimensional culture systems have been extensively studied for recapitulating chondrogenesis [3]. Among various approaches, injection of biomaterials loaded with BMSCs into the defect site is highly desirable for cartilage repair because such a procedure is micro-invasive and grafts are more flexible to fill the lesions with various shapes [4,5]. However, preventing the hypertrophic transition of BMSCs and morphological contraction *in vivo* still presents significant challenges in injectable graft [6].

The addition of multiple growth factors, such as transforming growth factor beta, insulin-like growth factor 1 and fibroblast growth factor 2, into the medium induces robust chondrogenesis of BMSCs *in vitro* [7,8], whereas differentiated BMSCs transplanted subcutaneously lead to hypertrophy and ossification replicating only the process of endochondral ossification [9]. In contrast, mature chondrocytes cultured within low passages regenerate cartilage with a stable phenotype ectopically. Further attempts to coculture BMSCs and chondrocytes in polymeric scaffolds revealed that chondrocytes induce chondrogenesis of BMSCs and prevent hypertrophic transition of differentiated BMSCs via secreting paracrine signals [10]. Additionally, cartilaginous ECMs produced by chondrocytes direct physical cell–matrix interaction and tether secretory growth factors with glycosaminoglycans, thus benefiting chondrogenesis of BMSCs [11,12]. Incorporation of chondrocytes into the injectable grafts is therefore a promising approach to construct the chondrogenic niche and enable the stable chondrogenesis of BMSCs.

Platelet-rich plasma (PRP) extracted from blood provides an autologous source of various growth factors; moreover, the incomparable biocompatibility and thrombin-stimulated clotting enabled PRP to be a promising cell carrier for tissue engineering [13]. Unfortunately, owing to poor mechanical stability and rapid degradability, direct mixing of chondrocytes with PRP leads to shrinking and deformed cartilage formation *in vivo* [14]. Combining chondrocytes and self-produced cartilaginous ECM during *in vitro* graft construction not only significantly enhances the morphological stability of grafts *in vivo*, but also carries multiple angiogenic inhibitors such as endostatin and chondromodulin I [15,16]; vascular infiltration-mediated ossification is therefore expected to be prevented. In a previous study we developed the cell bricks technique, which cultured a chondrocyte sheet and cut such a cell–ECM complex into multiple small fragments (cell bricks). We found that chondrocyte bricks significantly inhibited vascular infiltration into PRP gels and slowed their degradation, thus maintaining the framework and shape of the PRP grafts [17]. We hypothesized that the cell brick-enriched PRP gel could be an ideal injectable niche for BMSCs, which is expected to regenerate biological cartilage tissues with persistent cartilaginous phenotype, less deformation and uniform histological structure. In this study, we investigated the *in vivo* performance of BMSCs in cell brick-enriched PRP gels, and examined the mechanism of stable chondrogenic differentiation of BMSCs in such an injectable niche.

## Materials and methods

### Animals and experimental design

This animal experiment was approved by the Institutional Animal Care and Use Committee of the Fourth Military Medical University, Xi’an, PR China; the operative procedure and care of the mice were performed in accordance with the institutional guidelines of the committee. Forty-eight nude mice (6 weeks old, male, 24 to 28 g in weight) were used for the experiment. The mice were acclimated for 1 week before operation and monitored for general appearance, activity, excretion and weight. They were then randomly divided into three groups (*n* = 12 in each group): BMSCs–PRP (B-P) group, BMSCs–chondrocytes–PRP (B-C-P) group and BMSCs–cell bricks–PRP (B-CB-P) group. The animals were sacrificed for sample harvest at 4-week and 12-week time points (*n* = 6 at each time point). In addition, a chondrocytes–chondrocyte bricks–PRP (C-CB-P) group was used as a control group to evaluate the chondrogenesis through 12 weeks (*n* = 6), and a nasal augmentation group was used for functional tests (*n* = 6).

### Cell isolation

BMSCs were harvested and isolated from the tibiae and femora of 4-week-old New Zealand rabbits sacrificed by injecting overdoses of pentobarbital. After being physically disrupted, bone marrow was washed out from the fragments with 40 ml Dulbecco’s modified Eagle’s medium (DMEM; Hyclone, Logan City, Utah, USA) and centrifuged at 1,500 rpm for 5 minutes. The supernatant was removed and the cells were twice washed with phosphate-buffered saline (PBS) and then resuspended in DMEM low glucose supplemented with 10% fetal bovine serum (Hyclone), 50 μg/ml penicillin and 30 μg/ml streptomycin (Amresco, Cleveland, Ohio, USA). Primary cells were seeded in a 75 cm^2^ culture flask at 5 × 10^5^ nucleated cells/cm^2^ in DMEM low glucose supplemented with 10% fetal bovine serum (Hyclone), l-glutamine (272 μg/ml; Amresco), ascorbate-2-phosphate (50 μg/ml; Sigma, St. Louis, MO, USA), 50 μg/ml penicillin and 30 μg/ml streptomycin (Amresco) (medium I) and incubated at 37^○^C with 5% carbon dioxide. After 3 days, the nonadherent cells were removed during the first media change. Remaining adherent cells were further cultured with a medium change every 3 days. The adherent cells were incubated until cell clones reached over 80% confluence and then were digested with 0.25% trypsin (Hyclone) and subcultured at 1.0 × 10^4^ cells/cm^2^. Passage 1 cells were used for further experiments.

**Figure 1:**
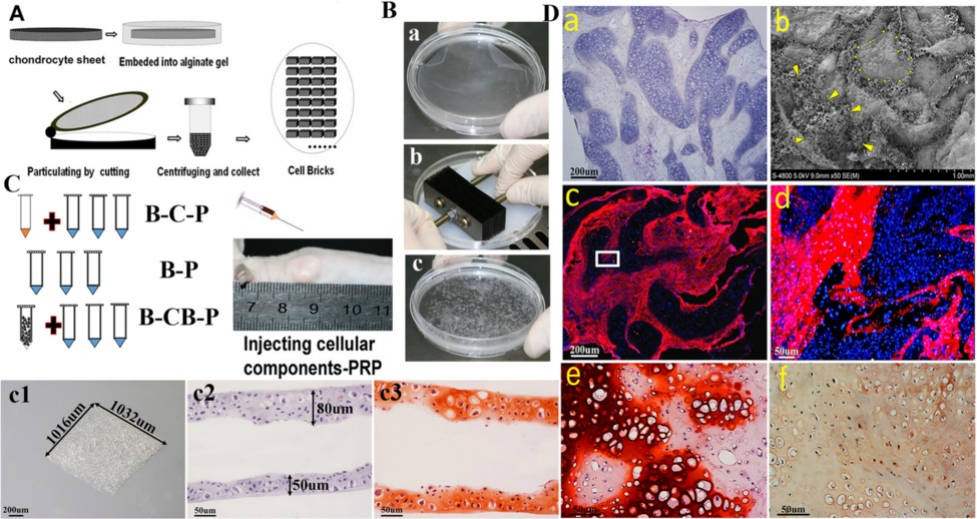
**Characterization of cell bricks and injectable cell brick–platelet-rich plasma gel. (A), (B)** Schematic description of the strategy for chondrocyte bricks and *in vivo* cell implantation. A chondrocyte sheet was cultured, harvested and embedded to be fragmented in a net cutting system (by multiple blades) (B.a, b, c). The thickness of the cell brick is about 50 to 80 μm; bar = 50 μm, magnification × 200 (B.c2, c3). Moreover, we used a stereomicroscope to measure the cell brick, which was 1,032 μm × 1,016 μm; bar = 200 μm, magnification × 30 (B.c1). **(C)** The obtained cell bricks or chondrocytes and cultured bone marrow-derived mesenchymal stem cells (BMSCs) mixed together or BMSCs alone were suspended in platelet-rich plasma (PRP), so that the injectable constructs were formed and injected subcutaneously into nude mice. B-C-P, BMSCs–chondrocytes–PRP; B-CB-P, BMSCs–cell bricks–PRP; B-P, BMSCs–PRP. **(D)** The graft maintained structural integrity at the fourth week postoperatively; bar = 200 μm, magnification × 40. (a) Scanning electron microscopy images showed BMSCs distributed evenly in the spaces formulated by chondrocyte bricks. (b) Even at the 12th week postoperatively, 5-bromo-2-deoxy uridine-labeled BMSCs (arrowheads) still occupied these spaces and further differentiated into tissues (c: low magnification, bar = 200 μm; d: high magnification, bar = 50 μm). Labeled BMSCs differentiated into newborn chondrocytes characterized by safranin-O staining and immunohistochemistry staining of collagen II (D.e, f).

Auricular chondrocytes were isolated from the ears of 4-week-old New Zealand rabbits as described previously [14]. Briefly, one-half of the primary chondrocytes were cultured in 25 cm^2^ culture flasks at 1.5 × 10^5^ cells/cm^2^ in medium I to enhance proliferation for 10 days. The other half of the isolated primary chondrocytes were cultured at 6.5 × 10^5^ cells/cm^2^ in six-well plates in DMEM high glucose supplemented with 20% fetal bovine serum (Hyclone), l-glutamine (272 μg/ml; Amresco), ascorbate-2-phosphate (50 μg/ml; Sigma), 50 μg/ml penicillin and 30 μg/ml streptomycin (Amresco) (medium II) and incubated at 37^○^C with 5% carbon dioxide. At day 10 when the solid white membrane of chondrocytes formed, the membranes were fragmented in a homemade cutting system in order to achieve cell bricks as described previously with some modification [17]. Briefly, after the cell sheet formed in the Petri dish, it was scratched and immersed in 1.2% alginate sodium and flattened. The solution of calcium chloride (102 mM) was then carefully dropped into the dish until the alginate solution turned into gel completely. The homemade cutting device is mainly composed of 20 multiple blades (Figure 1B), and the distance between each blade is 1 mm. The chondrocyte sheet–gel complex was then cut via compressing the cutting device vertically and horizontally. We always made gel 3 to 5 mm in thickness; then fragmented gel–cell complexes could be obtained without rolling up, and further were immersed into dissolving buffer (55 mM sodium citrate (Sigma) and 0.15 M sodium chloride (Sigma), pH 6.8) – thereafter, cell bricks were released.

### Preparation of platelet-rich plasma

Whole blood was aspirated from rabbit (New Zealand white rabbits weighing 2.5 to 3.0 kg) ventricle after anesthesia and was mixed with sodium citrate (3.8%) at a ratio of 9:1 for anti-coagulation. PRP was enriched by a two-step centrifugation process as described elsewhere. Briefly, 18 ml whole blood was drawn from the ventricle of each rabbit into two sterile tubes, each containing 1 ml sodium citrate (3.8%) solution as an anticoagulant. The tubes were then spun at 1,800 rpm for 8 minutes in a centrifuge at room temperature, and the blood separated into three phases: platelet-poor plasma (top), PRP (middle), and erythrocytes (bottom). The top and middle layers were transferred to new tubes and centrifuged again at 3,600 rpm for 8 minutes. The supernatant plasma was discarded, and the remaining 2 ml plasma containing precipitated platelets was blended evenly and designated PRP. The final platelet concentration was adjusted to 20.9 ± 1.1 × 10^8^/ml. PRP was preserved on ice for further steps.

### Preparation and transplantation of constructs

A sample of 500 μl PRP was used per animal. BMSCs, chondrocytes and cell bricks were collected and rinsed with PBS once. For the B-P group, BMSCs (2.25 × 10^7^ cells) were centrifuged and then suspended with PRP solution. For the B-C-P group, BMSCs (2.25 × 10^7^ cells) and chondrocytes (7.5 × 10^6^ cells) were centrifuged into a mixing pellet and resuspended with PRP sufficiently. For the B-CB-P group, BMSCs (2.25 × 10^7^ cells) and cell bricks (from 7.5 × 10^6^ primary cells) were centrifuged into a mixing pellet and resuspended with PRP sufficiently. For the C-CB-P group, chondrocytes (2.25 × 10^7^ cells) and cell bricks (from 7.5 × 10^6^ primary cells) were centrifuged into a mixing pellet and resuspended with PRP sufficiently. The injecting process was performed with the following procedure: PRP–cellular components were aspirated into a 2 ml syringe. The syringe was then aspirated with 50 μl thrombogenic agent (100 U/ml in 100 mg/ml calcium chloride; Villalba, Madrid, Spain Biomedical) and mixed. After being anesthetized with ethyl ether, the clotting constructs were injected subcutaneously into the dorsum of nude mice via a 16 G needle, and the construct turned into gel-like spheroid instantly after the mouse received it. An animal functional model was also constructed by subcutaneously injecting 300 μl B-CB-P components fabricated with same method as above into the nasal dorsum of nude mice. To characterize the cell bricks enriched with PRP gel, some pieces of complex were fixed in 2.5% glutaraldehyde, and then processed for scanning electronic microscopy (Jeol JSM6330F; Akishima, Tokyo Japan) examination.

### Gross morphology

After 4 weeks and 12 weeks, animals were euthanized and the samples were dissected from the surrounding tissues. Wet weights, thicknesses and volumes of the constructs were measured as described before [17]. Specimens were evaluated for contour deformation, including the constructs for functional experiment.

### Biochemical analysis (glycosaminoglycan quantification and collagen quantification)

Sulfated glycosaminoglycans (sGAGs) were determined for specimens using a Blyscan™ sGAG Assay Kit (B1000; Biocolor, Carrickfergus UK). sGAG was extracted from specimens by mincing the specimen into tiny pieces and digesting with papain extraction reagent (Sigma) at 65^○^C for 18 hours. The kit instructions were used to measure the sGAG concentration in the supernatant. sGAG per wet weight construct was calculated from the sGAG standard curve. Collagen content was quantified using a Sircol™ Collagen Assay (S1000; Biocolor). Collagen was extracted from specimens by mincing specimen segments into pieces of about 1 mm^3^ and digesting with pepsin (1 mg/ml suspended in 0.5 M acetic acid; Santa Ana, California, USA MP). The kit instructions were used to measure the collagen concentration in the supernatant. Total collagen per construct wet weight was calculated from the collagen standard curve.

### Histological and immunohistochemical assay

Samples were fixed in 4% paraformaldehyde and embedded in paraffin and then cut into 8 um sections. Sections were stained with hematoxylin and eosin (H & E), safranin-O, Masson trichrome or Von Kossa. To investigate the expression of type I, type II and type X collagen in matrices and vascular endothelial growth factor (VEGF), some sections were processed for two-step indirect immunohistochemical staining as studied previously [17]. Briefly, expression of type I collagen, type II collagen, type X collagen and VEGF was detected using primary anti-collagen type I antibody (mouse anti-rabbit, 1:50; Abcam, Cambridge, MA USA), anti-collagen type II antibody (mouse anti-rabbit, 1:50; Acris, Herford Germany), anti-collagen type X antibody (mouse anti-rabbit, 1:50; Abcam) and anti-VEGF antibody (mouse anti-rabbit, 1:50; Abcam), followed by horseradish peroxidase-conjugated anti-mouse antibody (1:200 in PBS; Santa Cruz Dallas, Texas, USA) and color development with diaminobenzidine tetrahydrochloride (Santa Cruz).

### BrdU and CD31 immunofluorescence assay

To determine the origin of new-born chondrocytes, 5-bromo-2-deoxy uridine (Brdu) was used to label BMSCs in the B-CB-P group. After 12 weeks, specimens were harvested and fixed in 4% paraformaldehyde overnight before creating frozen sections. The sections were permeabilized with 0.3% Triton X-100 at room temperature for 20 minutes. After being rinsed three times in PBS for 10 minutes, the samples were blocked with 5% goat serum for 20 minutes at room temperature. The samples were then incubated with primary anti-BrdU antibody (rat monoclonal, 1:100; Abcam) at 4°C overnight. After being rinsed three times in PBS for 10 minutes and incubated with Cy3-AffiniPure conjugated secondary antibody (goat anti-rat, 1:100; Jackson West Grove, PA America) at room temperature for 2 hours, the sections were incubated with DAPI for 5 minutes. Finally, the sections were washed three times in PBS for 10 minutes, covered with a coverslip and studied under a fluorescent microscope (Olympus IX71; Tokyo Japan).

The protocol of CD31 immunofluorescence assay for the B-CB-P group samples at 4 weeks and 12 weeks was the same as the Brdu immunofluorescence assay. Primary anti-CD31 antibody (mouse anti-rabbit, 1:100; Abcam) was incubated at 4°C overnight, followed by Cy3-AffiniPure conjugated secondary antibody (goat anti-mouse, 1:100; Jackson America).

### RNA isolation and real-time RT-PCR

Total RNA from rabbit auricular cartilage and different groups was extracted by RNAiso Plus (TaKaRa, Shiga Japan) followed by a one-step phenol chloroformisoamyl alcohol extraction, as described by the manufacturer’s protocol. Real-time RT-PCR analysis of five genes – VEGF, collagen (COL)-I, COL-II, COL-X and GAPDH – was performed using the One Step SYBR® PrimeScript™ RT-PCR Kit (TaKaRa)*.* The primer sequences used in this study are presented in Table 1. Real-time RT-PCR was replicated five times. The results are presented as target gene expression first normalized to GAPDH in the same sample (ΔCt), and then to the expression of that target gene measured in rabbit auricular cartilage as native control (ΔΔCt). The 2^–ΔΔCt^ method was used to compare differences of gene expression among the three groups.

**Table 1:** **Gene primer sequence for real-time RT-PCR**

**Gene**	**Primers**
COL-I	Forward 5′-GACATGTTCAGCTTTGTGGACCTC-3′
	Reverse 5′-GGGACCCTTAGGCCATTGTGTA-3′
COL-II	Forward 5′-GACCCCATGCAGTACATG-3′
	Reverse 5′-GACGGTCTTGCCCCACTT-3′
COL-X	Forward 5′-GGGATGCCTCTTGTCAGTGC-3′
	Reverse 5′-ATCTTGGGTCATAGTGCTGCTG-3′
VEGF	Forward 5′-ATCGAGACCTTGGTGGAC −3′
	Reverse 5′-CCTGGTGAGGTTTGATCC-3′
GAPDH	Forward 5′-TGGTATCGTGGAAGGACTCATGAC-3′
	Reverse 5′-ATGCCAGTGACGTTCCCGTTCAGC-3′

COL, collagen; GAPDH, glyceraldehyde 3-phosphate dehydrogenase; VEGF, vascular endothelial growth factor.

### Data analysis

All results are expressed as the mean ± standard deviation. Statistical analyses were performed using SPSS 17.0 software (SPSS, Chicago, IL, USA). Analysis of variance was used for multiple group comparisons, followed by Tukey’s honestly significant difference tests; *P* <0.05 was considered to indicate statistical significance.

## Results

### Characterization of cell bricks and injectable cell brick–platelet-rich plasma gel

At the end of the culture, the seeded chondrocytes produced sufficient ECM and transformed into semi-transparent white membrane (Figure 1B.a). After cutting with the homemade cutting device, square fragments of size 1 mm^2^ (Figure 1B.c1) as characterized by stereomicroscope were produced so that they could pass through a 16 G needle smoothly. For histological analysis of cell bricks, safranin-O staining was performed and showed that a large amount of glycosaminoglycan (GAG) occupied the extracelluar space, which confirmed formation of cartilaginous ECM (Figure 1B.c3). Before injecting cellular constructs, mixing cell bricks with PRP and then clotting resulted in morphologically stable blocks, which also efficiently maintained spheroid morphology subcutaneously after injection (Figure 1C). We further examined constructs 4 weeks after they were injected *in vivo* (Figure 1D). H & E staining showed chondrocyte bricks distributed evenly and supporting the constructs, spindle cells filling into the spaces among bricks (Figure 1D.a). Scanning electron microscopy images confirmed the observation from H & E images, and proved that chondrocyte bricks perform as a framework inside the PRP gel, and form multiple, enclosed cavities prepared for BMSC filling (Figure 1D.b). Brdu immunostaining confirmed that spindle cells filling in spaces among bricks were implanted BMSCs (Figure 1D.c, d); these cells consistently existed even after 12 weeks postoperatively, and presented as newborn chondrocytes characterized with safranin-O staining, thus constituting most of the newly formed cartilage among cell bricks (Figure 1D.e, f).

### Cell brick-enriched platelet-rich plasma gel guided chondrogenesis of BMSCs with minimal deformation and contraction

**Figure 2:**
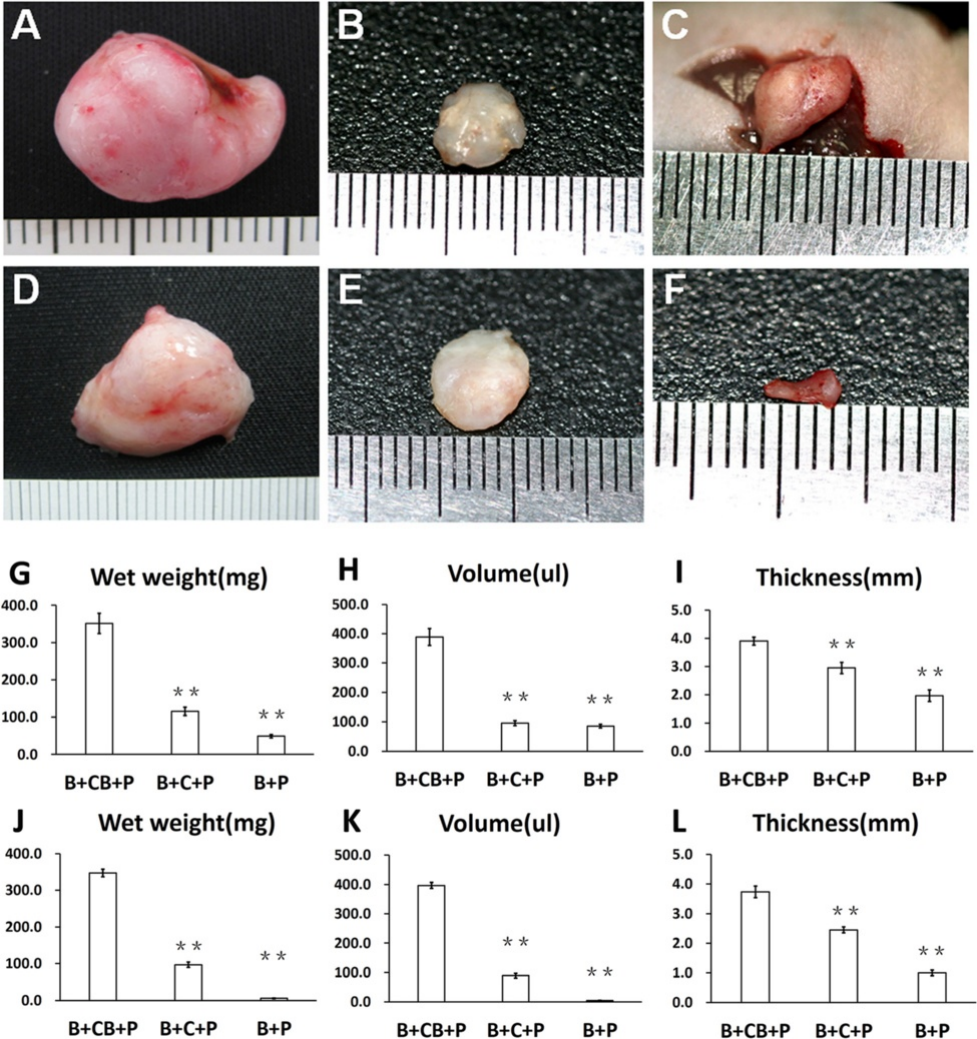
**Macroscopic examination of regenerated tissue form grafts.** Close macroscopic views of the regenerated cartilage from the B-CB-P **(A, D)**, B-C-P **(B, E)** and B-P **(C, F)** groups after 4 and 12 weeks of *in vivo* incubation. Samples harvested from mice at 4 weeks **(G, H, I)** and 12 weeks **(J, K, L)** postoperatively present different volume, weight and thickness. **P <0.01. B-C-P, BMSCs–chondrocytes–PRP; B-CB-P, BMSCs–cell bricks–PRP; B-P, BMSCs–PRP; BMSC, bone marrow-derived mesenchymal stem cell; PRP, platelet-rich plasma.

From the fourth week, observable knobbles could be detected in all animals. Gross morphology revealed that B-CB-P and B-C-P grafts transformed into tissues with white, pearly, opalescent appearance at both time points (Figure 2A,D), whereas the B-P group turned into vascularized and calcified tissues (Figure 2C). As compared with constructs injected instantly, the B-C-P and B-P groups contracted significantly after the fourth week (Figure 2B,C), characterized by compressed, irregular outline and uneven thickness throughout samples. In contrast, cell brick-enriched constructs (B-CB-P) significantly resisted *in vivo* contraction (Figure 2A). After 12 weeks, the B-CB-P group maintained their original morphology and presented a resilient property (Figure 2D), whereas B-C-P further turned into harder tissues and contracted more (Figure 2E). Moreover, the B-P knobbles were largely absorbed and almost disappeared subcutaneously at this time (Figure 2F). In order to quantitatively evaluate the roles of cell bricks in the resistance of graft shrinkage and deformation, we measured the volumes, thicknesses and wet weights of regenerated tissues after 4 weeks and 12 weeks postoperatively. As Figure 2G,J shows, wet weight measurement of all samples showed significant differences among groups (4 weeks: *n* = 6, *F* = 520.9, *P* < 0.0001; 12 weeks: *n* = 6, *F* = 3,601, *P* <0.0001). After 4 weeks, B-CB-P (351.7 ± 27.2 mg) was significantly larger than B-P (49.1 ± 4.7 mg, *P* <0.01) and B-C-P (115.2 ± 10.9 mg, *P* <0.01). After 12 weeks, B-CB-P (356.9 ± 10.2 mg) was significantly larger than B-P (5.6 ± 0.5 mg, *P* < 0.01) and B-C-P (96.7 ± 7.2 mg, *P* <0.01). Accordingly, volume measurements also indicated significant differences among the groups (4 weeks: *n* = 6, *F* = 572.6, *P* <0. 0001; 12 weeks: *n* = 6, *F* = 4,267, *P* <0. 0001). As Figure 2H shows, the volume of the B-CB-P group (388.6 ± 28.6 μl) was significantly higher than those of the B-P (85.6 ± 6.3 μl, *P* <0.01) and B-C-P (96.1 ± 8.5 μl, *P* <0.01) groups. After 12 weeks (Figure 2K), the volume of the B-CB-P group (396.6 ± 10.5 μl) was significantly higher than those of the B-P (5.0 ± 0.3 μl, *P* <0.01) and B-C-P (89.2 ± 8.3 μl, *P* < 0.01) groups. Moreover, the thickness of the samples are significantly different after 4 weeks (*n* = 6, *F* = 228.3, *P* <0. 0001) and after 12 weeks (*n* = 6, *F* = 549.0, *P* <0. 0001); at 4 weeks, the thickness of the B-CB-P group (3.9 ± 0.2 mm) was significantly higher than those of the B-P (2.0 ± 0.2 mm, *P* <0.01) and B-C-P (3.0 ± 0.1 mm, *P* <0.01) groups (Figure 2I). At 12 weeks (Figure 2L), the thickness of the B-CB-P group (3.7 ± 0.2 mm,) was significantly higher than those of the B-P (1.0 ± 0.1 mm, *P* <0.01) and B-C-P (2.5 ± 0.1 mm, *P* <0.01) groups. The above results suggest that cell bricks play an important role in the maintenance of morphology.

### Cell-bricks guided persistent ectopic chondrogenesis of BMSCs and prevented ossification

**Figure 3:**
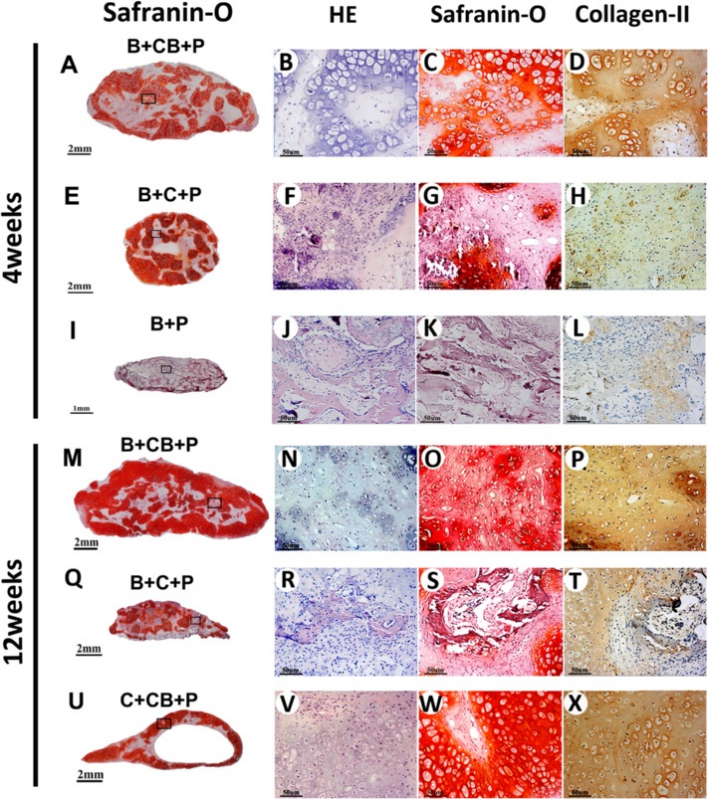
**Cell brick-enriched platelet-rich plasma gel guided chondrogenesis of bone marrow-derived mesenchymal stem cells.** Examining chondrogenesis of grafts *in vivo* at 4 weeks **(A to L)** and 12 weeks **(M to X)**. Merged images showed the B-CB-P and B-C-P groups acquired cell survival and tissue formation throughout the graft (A, E, M, Q), while the C-CB-P group accompanied central necrosis (U). The B-CB-P group presented chondrogenic differentiation in BMSC regions, as confirmed by hematoxylin and eosin (HE), Safranin-O and COL-II immunostaining (B, C, D; N, O, P), which is close to tissue formation from chondrocyte transplantation (V, W, X). In contrast, progressive osteogenesis occurred in the B-C-P and B-P groups (F, G, H; R, S, T; J, K, L); bar = 2 mm, magnification × 40 (A, E, M, Q, U); bar = 1 mm, magnification × 40(I); bar = 50 μm, magnification × 200 (B, C, D, F G, H, J, K, L, N, O, P, R, S, T, V, W, X). B-C-P, BMSCs–chondrocytes–PRP; B-CB-P, BMSCs–cell bricks–PRP; B-P, BMSCs–PRP; BMSC, bone marrow-derived mesenchymal stem cell; COL, collagen; PRP, platelet-rich plasma.

The cartilaginous characters of regenerated tissue were examined by safranin-O staining and type II collagen immunostaining, which further revealed different remodeling processes *in vivo* and different histological structures of engineered tissues. Following 4 weeks *in vivo*, in the B-P group BMSCs developed into cancellous bone (Figure 3I), and after 12 weeks these samples were almost completely absorbed (data not presented). We then used C-CB-P as a control group for evaluation of long-term chondrogenesis (Figure 3U). Samples from the B-CB-P and B-C-P groups presented tissue formation throughout the grafts, in contrast with central necrosis in the C-CB-P group, and cell survival and tissue development could be observed in the interior of these grafts. In B-C-P samples, two distinct tissue regions were presented: cell bricks derived cartilaginous tissue that was strongly positive for GAG staining, and BMSCs derived ossifying ECM (Figure 3E,F,G). Furthermore, 12 weeks of *in vivo* incubation resulted in cancellous bone and mature cartilaginous matrices from chondrocyte bricks. Surprisingly, no calcification and ossification were observed in samples from the B-CB-P group through 4 weeks of *in vivo* incubation. Instead, regions with typical cartilage morphology – that is, round cells with lacunae surrounded by ECM – were found sparsely distributed inside the formed tissues (Figure 3A,B,C,D). *In vivo* incubation through 12 weeks allowed merging of cartilaginous regions and emergence of cartilage shell in the B-CB-P group (Figure 3M); the BMSCs in this group continuously turned into round cells with lacunae surrounded by more cartilaginous matrix, which was characterized by deepening safranin-O staining and COL-II immunostaining (Figure 3N,O,P).

**Figure 4:**
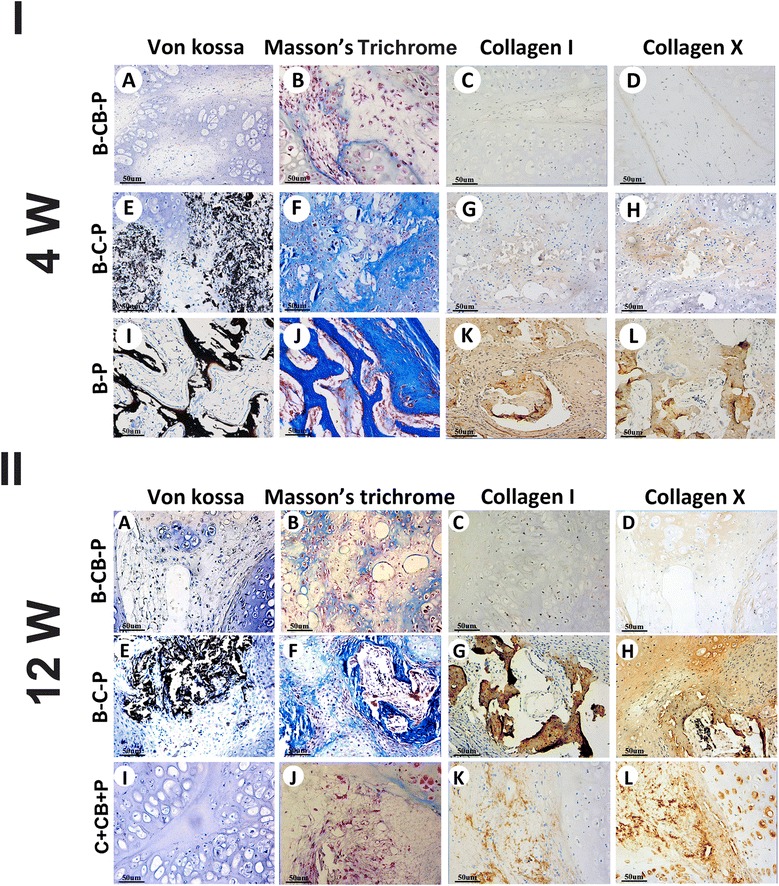
**Cell bricks guided persistent ectopic chondrogenesis of bone marrow-derived mesenchymal stem cells and prevented ossification.** Examining osteogenesis of grafts *in vivo* at 4 weeks **(I-A to I-L)** and 12 weeks **(II-A to II-L)**. The B-CB-P group did not present ossification of BMSCs through 12 weeks (I-A to D; II-A to D), as confirmed by Von-Kossa, Masson’s trichrome, COL-I and COL-X immunostaining, which is similar to the chondrocyte implantation group (II-I to L). In contrast, BMSCs in the B-C-P group presented slower calcification than the B-P group (I-E to H, I-I to L), whereas final ossification occurred through 12 weeks (II-E to H); bar = 50 μm, magnification × 200 (I-A to L, II-A to L). B-C-P, BMSCs–chondrocytes–PRP; B-CB-P, BMSCs–cell bricks–PRP; B-P, BMSCs–PRP; BMSC, bone marrow-derived mesenchymal stem cell; COL, collagen; PRP, platelet-rich plasma.

Just as Figure 4I-J,K,L shows, ossification is always an important issue that hinders application of BMSC ectopic chondrogenesis. Von-Kossa staining revealed that calcium shown as black-colored crystals deposited widely and hypertrophic transition rapidly occurred in the B-P group. Calcium deposits could also be found in the B-C-P group as early as 4 weeks postoperatively (Figure 4I-E), and significantly more calcium could be found at 12 weeks (Figure 4II-E), which confirmed osteogenesis of BMSCs in this group. Masson’s trichrome staining of samples demonstrated production of mature collagen in the B-C-P and B-P groups, which confirmed formation of mature bone (Figure 4I-F,J,II-F). In contrast, no calcium deposition and cartilaginous collagen production were shown in the B-CB-P group through 4 weeks and 12 weeks (Figure 4I-A,B,II-A,B), which is in accordance with the chondrocyte implantation group (Figure 4II-I,J) and thus presented chondrogenic performance. To further identify the hypertrophic transition of implanted BMSCs in different groups, type I and type X collagen immunostaining were performed. In accordance with histological examination, BMSCs in the B-C-P group started to express type I collagen and type X collagen around cells (Figure 4I-G,H) at the fourth week, and the newly formed ECM presented deeper staining for the above proteins through 12 weeks (Figure 4II-G,H), indicating that these cells underwent progressively hypertrophic transition. In contrast, BMSCs in the B-CB-P group maintained low-level staining of type I and type X collagen (Figure 4I-C,D;II-C,D), even after 12 weeks *in vivo*; the staining of both collagens was still significantly fainter than for B-C-P samples, which is similar with staining in the chondrocyte implantation group (Figure 4II-K,L).

**Figure 5:**
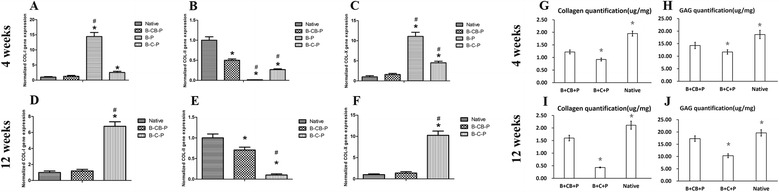
**Chondrogenesis-related gene expression and quantifications of glycosaminoglycan and collagen.** Real-time RT-PCR of samples at the fourth week **(A, B, C)** and 12th week **(D, E, F)** revealed the expression of Col-I, Col-II and Col-X in the cells of different groups which were all normalized to rabbit auricular cartilage. *Statistically significant difference from rabbit auricular cartilage (*P* <0.05). **#**Statistically significant difference from the B-CB-P group (*P* <0.05). **(G, H)** Quantitative analysis of collagen and sGAG content of constructs at the fourth week postoperatively. **(I, J)** Quantitative analysis of collagen and sGAG content of constructs at the 12th week postoperatively. *P <0.05, B-CB-P group versus B-C-P group, or native cartilage. B-C-P, BMSCs–chondrocytes–PRP; B-CB-P, BMSCs–cell bricks–PRP; B-P, BMSCs–PRP; BMSC, bone marrow-derived mesenchymal stem cell; Col, collagen; PRP, platelet-rich plasma; sGAG, sulfated glycosaminoglycan.

We monitored the transcript levels of Col-I, Col-II and Col-X of cells within the B-CB-P, B-C-P and B-P groups and native cartilage (Figure 5A,B,C,D,E,F). Figure 5B shows that the expression levels of Col-II genes in the B-CB-P group were significantly higher than in the B-P and B-C-P groups and even similar to native cartilage tissues (*P* <0.05). Furthermore, 12-week samples presented consistent Col-II expression in the B-CB-P group (Figure 5E). In contrast, hypertrophic markers such as Col-I and Col-X expression were significantly higher in the B-P and B-C-P groups through 12 weeks (Figure 5A,C,D,F; *P* <0.05). These results confirmed histological observations and suggested that chondrocyte bricks are more efficient in promoting chondrogenic differentiation of BMSCs than chondrocytes. Quantification of collagen and GAG within samples revealed significant differences among groups (4 weeks, *n* = 5: collagen, *F* = 210.5, *P* <0.0001; sGAG, *F* = 117.2, *P* <0.0001; 12 weeks, *n* =5: collagen, *F* = 36.54, *P* <0.0001; sGAG, *F* = 115.5, *P* <0.0001). After 4 weeks, the B-CB-P group presented the highest collagen content and GAG content (collagen, 1.22 ± 0.08 μg/mg; sGAG, 14.26 ± 1.26 μg/mg); although significantly lower than those in native cartilage (collagen, 1.95 ± 0.10 μg/mg; sGAG, 18.64 ± 1.66 μg/mg) (*P* <0.05), they were significantly higher than those in B-C-P group (collagen, 0.92 ± 0.06 μg/mg; sGAG, 11.68 ± 0.86 μg/mg) (*P* <0.05) (Figure 5C). After 12 weeks, the B-CB-P group presented the highest amount of collagen content and GAG content (1.60 ± 0.11 μg/mg; sGAG, 17.29 ± 1.13 μg/mg), although it is significantly lower than those in native cartilage (collagen, 2.11 ± 0.20 μg/mg; sGAG, 19.62 ± 1.31 μg/mg) (*P* <0.05), it was significantly higher than those in the B-C-P group (collagen, 0.43 ± 0.02 μg/mg; sGAG, 10.30 ± 0.82 μg/mg) (*P* <0.05) (Figure 5D).

### Angiogenesis regulated by cell bricks prevented central necrosis and ossification of constructs

**Figure 6:**
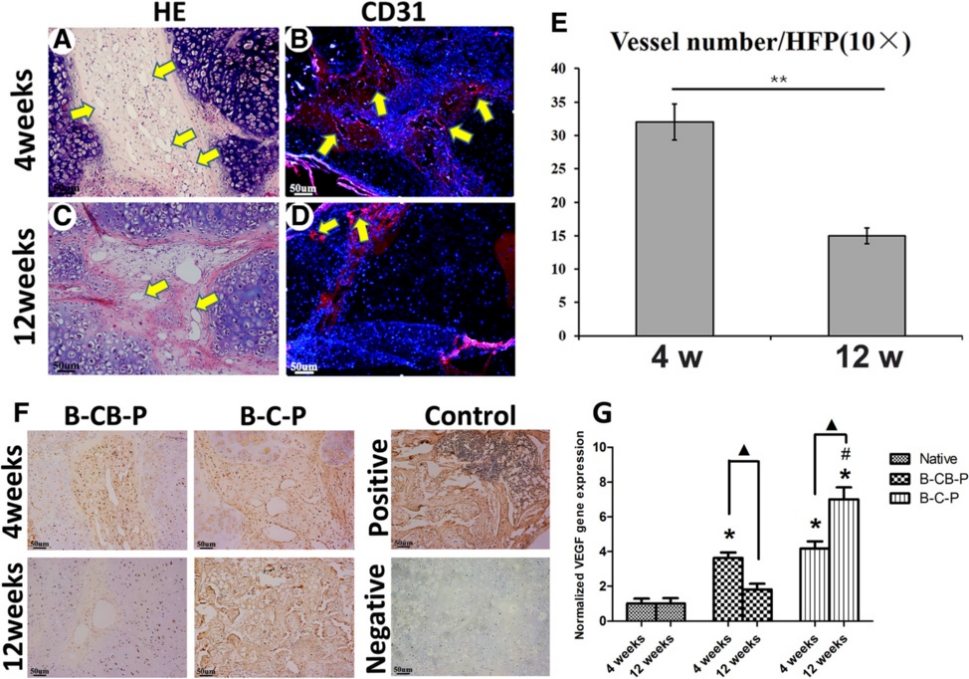
**Angiogenesis regulated by cell bricks prevented central necrosis and ossification of constructs.** Evaluation of angiogenesis in B-CB-P grafts at the fourth week and 12th week. **(A, C)** Histological images reveal infiltration of capillaries at the fourth week and 12th week, predominantly distributed in BMSC regions, as confirmed by CD31 immunofluorescent staining **(B, D)**. **(E)** Comparison of the number of blood vessels between the fourth week and 12th week as described previously; the value represents the cumulative number of all slides examined (*n* = 8). The fourth-week grafts allowed more blood vessel formation than those in the 12th week. **(F)** Immunostaining of vascular endothelial growth factor (VEGF) revealed decreasing expression of VEGF in BMSC regions through 4 and 12 weeks in the B-CB-P group, but increasing expression in the B-C-P group. Positive control, native cancellous bone; negative control, native auricular cartilage; bar = 50 μm, magnification × 100. **(G)** Real-time RT-PCR revealed the expression of VEGF in the B-CB-P and B-C-P groups, which were all normalized to rabbit auricular cartilage. *Statistically significant difference from rabbit auricular cartilage (*P* <0.05). **#**Statistically significant difference from the B-CB-P group (*P* <0.05). ▲Statistically significant difference of each group at two time points. B-C-P, BMSCs–chondrocytes–PRP; B-CB-P, BMSCs–cell bricks–PRP; B-P, BMSCs–PRP; BMSC, bone marrow-derived mesenchymal stem cell; HE, hematoxylin and eosin; HFP, high power field; PRP, platelet-rich plasma.

Nutritional perfusion determined survival of transplanted cells, especially for those in the interior of grafts. Histological examination revealed that cell brick-enriched PRP gel mixed with chondrocytes resisted graft contraction whereas it cannot support chondrocyte survival in the central part of grafts, thus causing central necrosis and formation of the central cavity (Figure 3U). Surprisingly, both B-C-P and B-CB-P grafts presented complete cellularization and tissue formation throughout grafts, but no central necrosis was observed in these samples. Especially for tissue formed in the B-CB-P group, both cell bricks and BMSCs maintained cell morphology and presented progressive chondrogenesis. Higher magnification displayed that circular, capillary-like tissues formed in BMSC regions of grafts as early as the fourth week. CD31 immunostaining (Figure 6A,B) confirmed that such a structure is constituted with endothelial cells, thus proving that BMSC regions permitted early vascular infiltration and nourished central tissues. Furthermore, we compared the number of blood vessels at the fourth week and 12th week (Figure 6C,D) by counting CD31-positive blood vessels and measuring their area according to a previously published procedure [18]. The average number of blood vessels at the fourth week was significantly higher than that of the 12-week group (32.0 ± 2.7 vessels/high power field vs. 15.0 ± 1.2 vessels/high power field, *P* <0.05) (Figure 6E). These data showed that the chondrogenic BMSCs resisted later hypertrophy and prevented angiogenesis-mediated endochondral ossification.

We further performed immunostaining and real-time RT-PCR to analyze expression of VEGF, which is known as a key component to induce angiogenesis. In accordance with angiogenesis in BMSC regions, the B-CB-P group and B-C-P group both expressed moderate VEGF since the fourth week (Figure 6F). Interestingly, VEGF was significantly decreased with longer *in vivo* incubation in the B-CB-P group (Figure 6F), which was completely different from endochondral ossification occurring in B-C-P samples (Figure 6F), and also explained why angiogenesis was inhibited through 12 weeks. Real-time RT-PCR confirmed the expression of VEGF in BMSC regions of constructs. Even though the expression of VEGF was significantly higher than that of native auricular, the expression of VEGF decreased in B-CB-P at the 12th week compared with the fourth week, which was just opposite to the results of the B-C-P group (Figure 6G, *P* <0.05). At the 12th week, BMSCs in the B-C-P group displayed higher expression of VEGF than B-CB-P samples (Figure 6G, *P* <0.05). Moreover, there was no significant difference in the expression of VEGF in the B-CB-P group and native auricular cartilage at the 12th week.

### B-CB-P grafts enabled stable nasal augmentation *in vivo*

**Figure 7:**
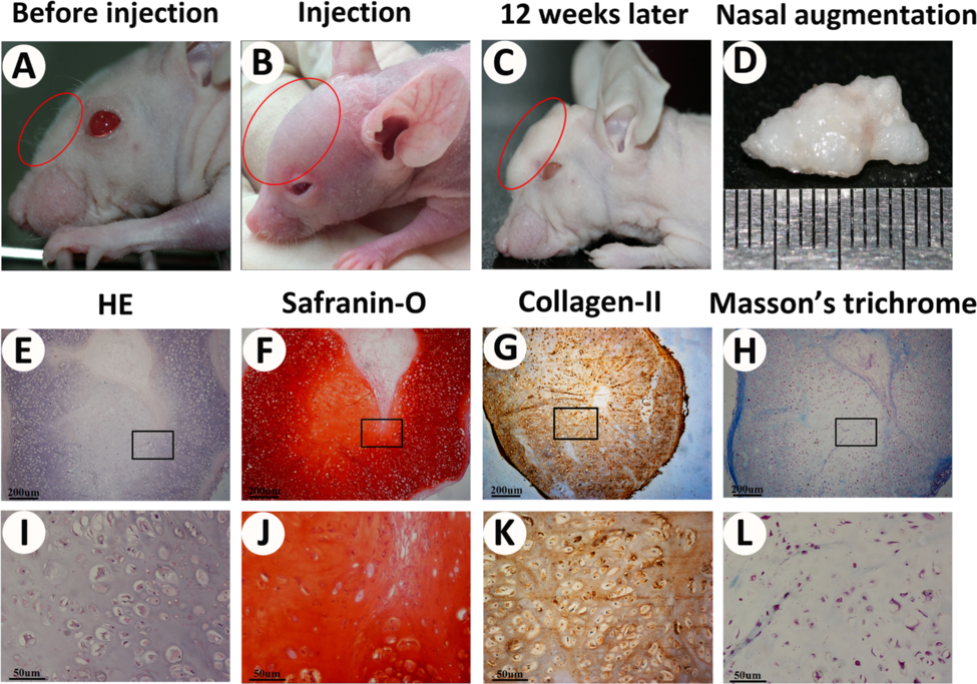
**B-CB-P grafts enabled stable nasal augmentation** ***in vivo.*** **(A to D)** Nasal augmentation regenerated by cell brick-enriched PRP gel composited with BMSCs. **(E to H)** Histological analysis including HE, safranin-O, Masson’s trichrome staining and COL-II immunostaining of the cross-sections further revealed that the samples formed homogeneous cartilage-like tissue with abundant lacunae as well as strong positive staining of GAG and type II collagen without ossification, and higher magnification for the above staining **(I to L)**; bar = 200 μm, magnification × 40 (E to H) and bar = 50 μm, magnification × 200 (I to L). B-CB-P, BMSCs–cell bricks–PRP; BMSC, bone marrow-derived mesenchymal stem cell; COL, collagen; HE, hematoxylin and eosin; PRP, platelet-rich plasma; GAG, glycosaminoglycan.

To test whether the B-CB-P complex could resist external pressure and could be applied for craniofacial reconstruction, nasal augmentation was performed using such an approach in nude mice. After 300 μl gel-like B-CB-P complex was injected subcutaneously into nasal dorsum, the skin was significantly elevated and gelling substances filled the subcutaneous cavity according to the customized nasal morphology. Slight volume reduction could be observed during the further *in vivo* remodeling process, which resulted in significant augmentation of the nasal dorsum (Figure 7A,B,C). After 12 weeks, the injected constructs turned into resilient, nasal ridge-like cartilage tissues (Figure 7D). H & E staining revealed BMSCs in constructs merged with chondrocyte bricks and most cells were embedded in a lacuna structure. Furthermore, safranin-O staining showed that most of constructs turned into glycosaminoglycan-enriched ECM, and COL-II immunostaining further confirmed that the type II collagen was widely expressed in ECM. Masson’s trichrome staining revealed no calcification and ossification were observed in samples (Figure 7E,F,G,H,I,J,K,L). These results demonstrated that B-CB-P grafts could be adapted into a customized morphology during the injecting process as well as later *in vivo* remodeling, therefore supporting regeneration of nasal cartilaginous tissue in craniofacial regions.

## Discussion

Developing an injectable approach for cartilage regeneration could meet today’s demand for micro-invasive surgery [19]. Different from solid scaffold-based tissue engineering, injectable cartilage constructs require fluidity of components and instant establishment of a chondrogenic niche for BMSCs, which is challenging for scaffold design. We have proved that fragmented chondrocyte macroaggregates (cell bricks) could stabilize the PRP gel efficiently *in vivo* and support chondrogenesis with stable morphology [17]. In this study, for the first time, we found that such an injectable complex could support chondrogenesis of BMSCs *in vivo*, and demonstrated that such a completely biological graft could meet the requirement of nasal augmentation, thus holding future promise in craniofacial reconstruction. The characterization of a niche constituted with cell bricks and PRP gel proved that brick-like chondrocytes–ECM complex could be smoothly injected and formulated PRP gel into multiple units; such enclosed spaces provided a chondrogenic niche for BMSCs. Further *in vivo* results revealed that the cartilaginous ECM in cell bricks provided adequate intrinsic resistance to surrounding pressure, and found that B-CB-P graft enabled morphological maintenance in a subcutaneous and even nasal dorsum environment, thus providing a novel and efficient strategy for cell transplantation. In contrast, mixing chondrocytes with BMSCs directly underwent significant contraction during *in vivo* remodeling, which could be ascribed to slow ECM formation and rapid degradation of PRP. These findings highlight the importance of cell bricks to establish injectable cell microenvironments for chondrogenesis of BMSCs *in vivo*.

Preventing ossification of BMSCs represents a critical step towards clinical translation of BMSCs in cartilage tissue engineering [20]. The most important finding in this study is that BMSCs embedded in chondrocyte brick-enriched PRP gel underwent persistent chondrogenesis, and hypertrophic translation was prevented. Coculturing mature chondrocytes and BMSCs has been shown to significantly reduce hypertrophic potential of BMSCs and support neocartilage development *in vivo* [21]. Paracrine signaling of soluble chondrogenic factors provided by chondrocytes was an important mechanism in directing the *in vivo* ectopic chondrogenesis of BMSCs [10,22]. It is also known that cartilaginous ECM contains important anti-angiogenic factors such as endostatin and chondromodulin I [15,16]. Previous coimplantation of BMSCs and chondrocytes frequently requires solid scaffold-based cell incubation prior to *in vivo* transplantation, so that cartilaginous ECM could be produced to constitute the enclosed niche for BMSCs before *in vivo* remodeling was initiated [23]. Interestingly, we found direct mixing of chondrocytes and BMSCs with PRP could delay the endochondrial ossification of BMSCs, whereas it failed to prevent final ossification. *In vivo* results showed that endochondral ossification started from BMSCs adjacent to surrounding host tissues. We suspected that the opening microenvironment caused by fast degradation of peripheral PRP failed to retain paracrine factors released from chondrocytes, which resulted in progressive ossification of BMSCs. Different from the opening structure in the B-C-P group, chondrocyte bricks reconstituted PRP into multiple cavities and further merged into enclosed ECM shell for graft at the 12th week; such an enclosed cartilaginous microenvironment definitely protected BMSCs from intervention surrounding the host microenvironment and activation of endochondral ossification. This finding warranted reduction of the donor cartilage as compared with conventional chondrocyte transplantation, and provide a micro-invasive approach for cartilage regeneration, indicating the promising future of cell bricks for clinical application. For clinical translation, donor cartilage could be harvested from the nasal septum, part of the auricle, nonloading articular surface and residual cartilage from microtia. Additionally, we are exploring the possibility of acellular chondrocyte sheets in inducing chondrogenesis of BMSCs, so that heterogeneous chondrocytes could be used for achieving cell bricks that may release donors thoroughly in the future.

Blood vessel invasion is a requisite aspect of endochondral ossification; on the other hand, adequate vascularization is critical for nutritional perfusion and tissue survival [18,24]. The important advantage of PRP gel is that it could initiate faster tissue remodeling than synthetic polymers, and simulate the wound healing process. A surprising phenomenon presented in our study is that BMSCs mixed into CB-PRP gel guided fast angiogenesis in BMSC regions and prevented central necrosis of the whole graft. Our previous experiment encountered the problem that chondrocytes mixed into CB-PRP gels resulted in obvious necrosis in the interior of constructs, which could be attributed to the robust anti-angiogenic potential of chondrocytes [20,25]. BMSCs, especially those stimulated by a hypoxia environment, convey essential angiogenic signals including VEGF, matrix metalloproteinase 9, stromal cell-derived factor 1 and granulocyte macrophage colony–stimulating factor [26-28], and VEGF is the most important mediator of angiogenesis, which couples recruitment of endothelial cells, capillary infiltration and ossification of hypertrophic cartilage [29-31]. Our results confirmed that BMSCs in CB-PRP gels presented higher VEGF expression than chondrocytes in CB-PRP gels, which was regarded to contribute the angiogenesis in BMSC regions in PRP grafts. This finding also indicated that B-CB-P grafts are superior to the previous cell–solid scaffold approach because they avoided the poor nutritional perfusion in central regions. Furthermore, our study revealed that the early angiogenesis of BMSC regions in B-CB-P grafts did not resulted in hypertrophy of chondrogenic BMSCs. The C-CB-P group in our previous study [17] and the B-CB-P group in this study both expressed type I and type X collagen; however, expression of these proteins was at a very low level. In this study, real-time RT-PCR confirmed that the gene expression of type I and type X collagen in the B-CB-P group was obviously lower than other groups, which demonstrated the inhibited hypertrophy in this group. From 4 to 12 weeks, B-CB-P grafts presented decreasing VEGF, and the amount of capillaries significantly decreased in BMSCs with the chondrogenic differentiation of BMSCs. In contrast, progressive ossification of BMSCs in B-C-P and B-P grafts presented hypertrophic makers such as Col-X and increasing vascularization. Although the further mechanism remained to be unraveled, this finding indicated that chondrocyte bricks interrupted angiogenesis-mediated endochondral ossification, which played a key role in realizing the persistence of chondrogenic differentiation of BMSCs.

GAG and collagen production are related to the mechanical properties of cartilage. In our study, although the 12-week results presented cartilage formation in BMSC regions, safranin-O staining and COL-II staining indicated that the cartilaginous matrices formed by chondrogenic BMSCs are still less than those of chondrocytes. Quantification of the ECM also confirmed our observation. These results indicated that chondrogenic differentiation of BMSCs in B-CB-P grafts is still insufficient. Owing to the lifespan of the nude mice, whether longer *in vivo* development will allow better chondrogenesis remains to be investigated. Further study will use autologous cell transplantation in a rabbit model so that long-term results for 6 months to 1 year could be evaluated. The other possible reason for insufficient chondrogenesis may be too much distance between BMSCs and chondrocyte bricks. Different from direct mixing of cells, gaps between BMSCs and chondrocyte bricks will weaken the paracrine signals released from cell bricks. The size of cell bricks should therefore be optimized further in studies so that adequate distribution could be acquired.

## Conclusion

The findings reported here provided a novel, efficient and injectable approach to regenerate cartilage tissues *in vivo*. Compared with chondrocytes, chondrocyte bricks could maintain the graft morphology *in vivo*, enable cartilage repair with specific shape, and persistently prevent hypertrophic transition of BMSCs *in vivo*. On the other hand, BMSCs interacted with cell bricks and mediated early vasculogenesis in the interior of graft, which warranted the survival of interior cells and chondrogenesis of whole grafts.

## Abbreviations

BMSC: bone marrow-derived mesenchymal stem cell
Brdu: 5-bromo-2-deoxy uridine
COL: collagen (gene)
DMEM: Dulbecco’s modified Eagle’s medium
ECM: extracellular matrix
GAG: glycosaminoglycan
GAPDH: glyceraldehyde 3-phosphate dehydrogenase
H & E: hematoxylin and eosin
PBS: phosphate-buffered saline
PRP: platelet-rich plasma
sGAG: sulfated glycosaminoglycan
VEGF: vascular endothelial growth factor
